# Ex-ante reminders: The effect of messaging strategies on reducing non-sustainable consumption behaviors in access-based services

**DOI:** 10.3389/fpsyg.2022.984222

**Published:** 2022-09-06

**Authors:** Xiaorong Fu, Yang Xu

**Affiliations:** School of Business Administration, South Western University of Finance and Economics, Chengdu, China

**Keywords:** access-based services, consumer non-sustainable consumption behaviors, message appeal strategy, message framing strategy, psychological ownership

## Abstract

Users’ non-sustainable consumption behaviors are affecting the sustainability of access-based services (ABSs), but ABS firms can utilize messaging strategies (ex-ante reminders) to persuade users to curtail their non-sustainable consumption behaviors. Through two online scenario-based experiments in China, this study determined that: (1) Compared with rational appeal messaging, emotional appeal messaging is better able to persuade consumers to curtail non-sustainable consumption behaviors. Furthermore, loss-framed messages are more effective than gain-framed ones. (2) Message appeal and message framing have an interactive persuasive effect on reducing such consumer behaviors. Loss-framed rational appeal messages are more persuasive at reducing non-sustainable consumption behaviors than gain-framed rational appeal messages, and gain-framed emotional appeal messages persuade consumers to reduce non-sustainable consumption behaviors more than loss-framed ones. (3) Consumers’ psychological ownership moderates the persuasive effect of messaging. Among consumers with a high level of psychological ownership of shared goods, only gain-framed emotional appeal messaging is effective at reducing non-sustainable consumption behaviors, whereas among consumers with low psychological ownership, the persuasive effect of loss-framed rational appeal messaging is more effective than gain-framed emotional appeal messaging. This study extends the research on non-sustainable consumption behavior management in ABSs and provides important inspiration for the management of ABSs consumer behavior.

## Introduction

Access-based services (ABSs) enable consumers to access goods, physical facilities, networks, labor and space while avoiding the hassle and expense of legal ownership (Schaefers et al., 2016a; Guo and Lamberton, 2021), such as bike-sharing, car-sharing, the P2P accommodation, etc. But consumers non-sustainable consumption behavior (e.g., tearing up QR codes, picking bike locks, and vandalism) is quite common in ABSs (Schaefers et al., 2016a; Jia et al., 2018; Kim, 2018; Ma et al., 2020), it even hinders sustainable development of the industry (Gerstlberger et al., 2014; Dangelico and Vocalelli, 2017; White et al., 2019).

Since digital sharing platforms in ABSs are able to deliver messages to consumers *via* mobile apps, new media, etc. (Belk, 2014; Piscicelli et al., 2015; Hamari et al., 2016; Jia et al., 2018; Basili and Rossi, 2020), messaging may becomes one of the easiest and most cost-effective methods of inducing changes in consumer non-sustainable consumption behavior. On the one hand, people often go to great lengths to conceal their non-sustainable consumption behaviors making it difficult for ABS firm managers to detect such behaviors while they are being used (e.g., supervising consumers’ use of goods) or afterwards (e.g., punishing individuals for misbehaving; Schaefers et al., 2016a). On the other hand, some firms have sought recourse in ex-ante governance by using targeted messaging strategies to deliver key information before consumers use shared goods for precautionary vigilance and persuasion, such as the P2P accommodation platform Xiaozhu uses rule information to prompt customers to stay civilized (Ma et al., 2020).

Meanwhile, researchers theorize that “who, what, why, how, and where” are key factors affecting the effectiveness of message dissemination (Lasswell, 2007). When the disseminators, audience, and communication channels are relatively fixed, the content of a message (what is said) and the message structure (how it is said) become the key factors that determine the effectiveness of a message. From the perspective of message content, firms’ ex-ante reminders generally achieve the purpose of alerting consumers through educational content or deterrence content (Fullerton and Punj, 2004). Educational content aims to suppress non-sustainable consumption behaviors by activating consumers’ internal recognition of social values and depends on both parties’ emotional resonance (Giorgi, 2017). Deterrence restrains non-sustainable consumption behaviors by guiding consumers to calculate actual benefits and is affected by the audience’s rational perceptions (Wu and Wang, 2011). We theorize that emotional appeal messaging and rational appeal messaging can achieve their respective aims of educating and deterring consumers. Specifically, based on previous research on message appeals (Antonetti and Maklan, 2014; Koenig-Lewis et al., 2014; Matthes et al., 2014), we propose that rational appeal messaging refers to messages that convey behavioral norms and behavioral consequences to individuals based on formal rules to achieve behavioral persuasion (Matthes et al., 2014). And emotional appeal messaging conveys a group’s attitude toward behaviors and informal social norms to the individual, stimulates the individual’s positive or negative emotional state, and then guides the individual’s behavior (Antonetti and Maklan, 2014; Koenig-Lewis et al., 2014). Loss framing and gain framing are two different ways of structuring messages (Kahneman and Tversky, 1979). Loss framing emphasizes the cost of doing something and reflects the negative results of an action (Kao, 2012), such as penalties for consumers who violate rules, while gain framing emphasizes the benefits that can be obtained by doing something and reflects the positive results that can be achieved by working hard (Kao, 2012), such as benefits (rewards) for following the rules.

Researchers have examined the independent influence of message appeal and message framing strategies on audience behavior. Some scholars determined that emotional appeals and rational appeals have different effects on influencing audience’s opinions and behaviors (e.g., Cutler et al., 2000; Huertas and Campomar, 2009; Antonetti and Maklan, 2014; Koenig-Lewis et al., 2014; Matthes et al., 2014; De Veirman et al., 2015; Keshari and Jain, 2016). Scholars also agree that different message framings have different persuasive effects (e.g., Meyerowitz and Chaiken, 1987; Block and Keller, 1995; Levin et al., 1998; Gallagher and Updegraff, 2012; McKinley et al., 2013; Macapagal et al., 2017; Ye et al., 2021). However, most of these research conclusions were derived from traditional consumption scenarios such as pharmaceuticals, electronics and hotels, etc. (e.g., Gerend and Shepherd, 2007; Zhao and Pechmann, 2007; Huertas and Campomar, 2009; Lin, 2011; Lwin et al., 2014; Ye et al., 2021). Few studies incorporate them into ABS scenarios for verification. To our knowledge, only the research of Zhang et al. (2020) considers the influence of message appeal on consumers’ willingness to participate in access-based consumption.

In ABSs, the lack of legal ownership makes users less responsible for maintaining goods, which makes non-sustainable behavior more likely to occur (Jussila et al., 2015; Fritze et al., 2020; Peck et al., 2021). In addition, in this consumption scenario, an ABS’s platform operation mode limits the firm’s timely monitoring of non-sustainable behaviors (Schaefers et al., 2016a). Scholars have suggested that psychological ownership as an alternative to legal ownership has a twofold impact on behavior. Greater psychological ownership may prompt consumers to proactively maintain goods (Jussila et al., 2015; Peck et al., 2021), but also stimulate territorial responses which may lead to negative consumer behavior, such as negative (non) verbal displays or other behavioral resistances, etc. (Kirk et al., 2018). Thus it is worthwhile to explore messaging strategy as a way to manage and control non-sustainable consumption behaviors and the role of psychological ownership in ABSs.

Therefore, we put forward the following research questions:

RQ1: When consumers do not have legal ownership of goods in ABSs, do messaging strategies incorporating different message appeals and message framings persuade them to curtail non-sustainable consumption behavior?

RQ2: Are there differences in the persuasive effects of these messaging strategies incorporating different message appeals and message framings?

RQ3: Does psychological ownership of shared goods have any influence on persuasive effects?

Through two online scenario-based experiments in China, this research answers to these questions. And the findings enrich current research on ABSs (Fritze et al., 2020) and provide a theoretical basis for ABS firms’ governance of non-sustainable consumption behavior.

## Literature review

### Non-sustainable consumption behavior in ABSs

ABSs are defined as services that allow customers to access a good, physical facility, network, labor, or space for a defined period of time, in return for an access payment, while legal ownership remains with the service provider (Lovelock and Gummesson, 2004; Bardhi and Eckhardt, 2012; Schaefers et al., 2016a,b), such as car and bike sharing (e.g., Zipcar), short-term rentals of fashion items (e.g., Bag Borrow), or peer-to-peer platforms (e.g., AirBnB; Srivastava et al., 2021). And ABSs have also been referred to as sharing (Belk, 2014), collaborative consumption (Bardhi and Eckhardt, 2012) or access-based consumption (Jin et al., 2020).

Existing research on ABSs generally assumes that consumers behave well (Xie and Mao, 2017). However, this is inconsistent with the documented frequency of bad behavior on sharing platforms, Ma et al. (2020) pointed out that there are non-sustainable consumption behaviors in various ABSs, such as in Airbnb (Kim, 2018), bike-sharing (Jia et al., 2018), car-sharing (Schaefers et al., 2016a), etc. Unfortunately, non-sustainable consumption behavior negatively affects the ABSs firms’ profitability and operational viability (e.g., damaging company property), it can also ruin the experience of other consumers (Verhoef et al., 2009). Although ABSs are intended to co-create consumer value through consumer participation in value creation, non-sustainable consumption behavior disrupts the service experience for other consumers (Plé and Cáceres, 2010; Yin et al., 2019) and lead to value co-destruction (Cabiddu et al., 2019; Yin et al., 2019). In this study, non-sustainable consumption behavior is defined as improper use of shared goods, such as vandalism, illegitimate possession, opportunism, etc. (Schaefers et al., 2016a; Jia et al., 2018). These behaviors are also known by various names such as misbehavior (Schaefers et al., 2016a; Jin et al., 2020; Srivastava et al., 2021), uncivilized behaviors (Jia et al., 2018), dysfunctional behaviors (Harris and Reynolds, 2003), etc.

In ABSs, the fact that consumers do not have legal ownership of goods makes unsustainable behaviors prone to occur, and governance of these behaviors is difficult for several reasons. First, consumers find many ways to conceal their non-sustainable consumption behaviors and make them difficult to detect and monitor. Consequently, most such consumption goes unnoticed (Lin et al., 1994; Lindblom and Kajalo, 2011). Shared goods that are mobile, such as cars or bikes, are especially difficult to monitor in real time. For example, shared cars can be picked up, driven and returned without service provider supervision, making it difficult to monitor user behavior or assess the condition of a returned car (Schaefers et al., 2016a). Second, platform consumers’ non-sustainable consumption behaviors show great externality. Since ABSs are based on users’ temporal use of shared goods within a specified period of time, a previous user’s non-sustainable consumption behavior affects other users’ subsequent use of those shared goods. Belk (2010) emphasized that the misbehavior of irresponsible users disrupts the use of the same shared good by subsequent users. Furthermore, compared with traditional services, the likelihood of non-sustainable consumption behavior contagion is higher among ABSs consumers. Studies have found that there is a significant connection between the misbehavior of previous users and future users’ misbehaving intentions (Schaefers et al., 2016a; Srivastava et al., 2021). Third, consumers’ non-sustainable consumption behaviors are affected by their psychological ownership of the shared goods. With the ABS model, consumers do not have legal ownership of goods (Fritze et al., 2020; Morewedge et al., 2021), but their psychological ownership has a strong impact on their consumption behavior (Jussila et al., 2015; Kirk et al., 2018).

Management of consumer behavior aims to ensure that platform users behave appropriately (Fullerton and Punj, 2004). In the ABS research field, existing research on platform governance tends to focus on the issue of platform system management from a macro perspective (Martin et al., 2017; Vith et al., 2019). Research on consumer behavior examines how to attract consumers and/or explores the antecedents of consumers’ appropriate or inappropriate behavior. There is a paucity of research on effective governance of users’ non-sustainable consumption behaviors. For example, Ning and Hu (2022) studied the positive influence imposed by social support of online travel platform enterprises in sharing economy on customer citizenship behavior. Fritze et al. (2020) proposed that psychological ownership can replace legal ownership as a way to encourage participation in ABSs. Jia et al. (2018) found that social norms and customer interface design are factors that contribute to user misbehavior in bike-sharing services (such as Mobike). Wang et al. (2019) suggest that social influences exert a positive impact whereas economic factors (i.e., pricing) have a negative influence on sustainable consumption behaviors. Ma et al. (2020) noted that in the peer-to-peer (P2P) accommodation industry, interpersonal trust, property experience and platform governance are important antecedents for customer civility. In addition, Schaefers et al. (2016a) and Srivastava et al. (2021) unraveled misbehavior contagion in ABSs.

Since non-sustainable consumption behavior is widespread among ABSs and its negative impact is objectively present, our study intends to explore the management mechanism of firms messaging strategies on users’ non-sustainable consumption behavior in order to reduce its negative impact on ABSs.

### Messaging strategies and non-sustainable consumption behavior

Liu et al. (2009) point out that it is possible to reduce non-sustainable consumption behaviors by conveying corporate values, accepted ethical and social norms, or behavioral guidelines in the process of service design and delivery. Given users’ penchant for concealment of their non-sustainable consumption behaviors, a platform’s focus on governance of such behaviors leans toward ex-ante governance, such as pre-education and message reminders. However, digital sharing platforms (Belk, 2014; Piscicelli et al., 2015; Hamari et al., 2016) rely on convenient information exchange through mobile apps, new media, etc. (Jia et al., 2018; Basili and Rossi, 2020). Therefore, ex-ante governance of consumer behavior is embedded in the delivery of information to users in order to activate precautionary vigilance and persuasion.

Firms’ ex-ante reminders generally achieve the purpose of alerting consumers through educational content or deterrence content (Fullerton and Punj, 2004). The educational approach uses promotional messages to instill negative attitudes about inappropriate behavior and strengthen moral constraints which inhibit such behavior. Effectiveness depends on both parties’ emotional resonance (Giorgi, 2017), and so message content needs to use emotional appeal (Antonetti and Maklan, 2014; Koenig-Lewis et al., 2014) to evoke positive or negative emotions in consumers in order to achieve emotional resonance. The deterrence approach presents information to make consumers understand potential consequences and reduce their intention to misbehave (Dootson et al., 2018). For example: shop-lifting is a crime (Dootson et al., 2017). The message content must convey behavioral norms and consequences based on formal rules to be effective; thus it is a message based on rational appeal (Matthes et al., 2014). Theoretically, firms can use emotional appeal messages to achieve consistency with public perceptions of non-sustainable consumption behavior, thereby persuading users to curb such behaviors. Firms can also use rational appeal messages to deter non-sustainable consumption behaviors. For example, the P2P accommodation platform Xiaozhu informs customers that if they violate platform use rules, a property owner has the right to deduct damages from their deposit (Ma et al., 2020).

From the perspective of messaging structure, scholars have shown that different framing techniques elicit different persuasive effects (Meyerowitz and Chaiken, 1987; Block and Keller, 1995; Levin et al., 1998). For example, positive *vs*. negative message framing affects acceptance of the message (Levin et al., 1998). Given that a message can frame consumer behavioral consequences as negative loss or positive gain, we refer to the presentation frame of message content as loss framing or gain framing (Kahneman and Tversky, 1979). Specifically, gain-framed messages emphasize the benefits that can be obtained by doing something and the positive results that can be achieved by hard work, while loss-framed messages emphasize the cost of doing something and the negative result of a behavior (Kao, 2012).

Previous studies have found that there are differences in the impact of emotional appeals and rational appeals on consumer behavior, some scholars have found that emotional appeals are more effective than rational appeals on consumers’ response (e.g., Cutler et al., 2000; Okazaki et al., 2010, 2013; Lwin et al., 2014; Zarantonello et al., 2014; De Veirman et al., 2015; Wei et al., 2015; Zhang et al., 2020), while other scholars have found the opposite (Huertas and Campomar, 2009; Lin, 2011; Keshari and Jain, 2016). Simultaneously, scholars have also identified boundary conditions to reconcile conflicting findings of message appeal, such as self-regulatory focus (Cornelis et al., 2012), product involvement (Holmes and Crocker, 1987), processing style (Ruiz and Sicilia, 2004), social norms (Zanon and Teichmann, 2016), and service types (Zhang et al., 2014), etc. Similarly, previous studies have also determined that the influence of message framing on consumer behavior has boundary effects, such as individual involvement levels, cognitive needs, and emotional characteristics (e.g., Block and Keller, 1995; Zhang and Buda, 1999; Donovan and Jalleh, 2000). Considering the persuasive differences of message appeals and message framings (e.g., Gerend and Shepherd, 2007; Zhao and Pechmann, 2007; Huertas and Campomar, 2009; Lin, 2011; Lwin et al., 2014; Ye et al., 2021), as well as the differences in consumers’ psychological ownership of ABS goods (Bardhi and Eckhardt, 2012; Fritze et al., 2020), this study explores the effect and mechanism of different message appeals and message framings on reducing non-sustainable consumption behavior and how users’ psychological ownership influences this effect and mechanism.

### Psychological ownership in ABSs

Psychological ownership refers to a personal sense of possession an individual holds for a good or part of a good which is not owned by the individual (Pierce et al., 2003). In ABSs, users’ psychological ownership is an important substitute for legal ownership (Fritze et al., 2020). As users come to intimately know, control, and invest themselves in shared goods through labor (Belk, 1988; Pierce et al., 2001) they may generate psychological ownership of the shared good. An individual’s psychological ownership increases (Peck and Shu, 2009) with long-term use and touch of the object (Strahilevitz and Loewenstein, 1998).

Psychological ownership has various effects on cognition and behavior. It can generate positive effects on consumer behavior, such as long-term customer loyalty, positive word-of-mouth, greater satisfaction, and more active participation in the protection and maintenance of an object (Jussila et al., 2015; Peck et al., 2021). For example, tenants may develop psychological ownership of an apartment in which they have lived for a long time and take the initiative to maintain the apartment. In terms of negative effects, psychological ownership can arouse territorial awareness and produce a negative response to other individuals who display ownership signals relative to the same object (Kirk et al., 2018).

Use patterns, such as the duration of use of a shared good, also impact psychological ownership (Strahilevitz and Loewenstein, 1998). Usage can affect a consumer’s response to messaging strategies and the persuasive effect on non-sustainable consumption behavior. Therefore, it is worthwhile to explore the influence of psychological ownership in the context of message strategies that affect non-sustainable consumption behavior.

## Hypotheses development

### Effect of message appeal on reducing non-sustainable consumption behavior

Rational appeal messages are based on formal rules that guide behavior based on actual gains and losses (Zhang et al., 2014). The effect of behavioral rules that emphasize actual gains and losses on consumer behavior depends on the individual’s perception of enforceability. Deterrence theory suggests that rule-based rational appeal messaging relies on rule enforcement certainty for persuasive effect (Fullerton and Punj, 2004). Because it is difficult for ABSs to monitor user behavior in real time, consumers perceive that rule enforceability is weak, and thus rational appeal messaging aimed at restraining non-sustainable consumption behaviors are largely has limited persuasive effect. Yao et al. (2019) have shown that user policies based on the credit system and formal sanctions for violations in bike-sharing firms did little to inhibit bad behavior. By contrast, informal social controls, such as social norms, have a deterrent effect on unethical behavior (Albers-Miller, 1999) by increasing consumers’ perceptions of the likelihood of being caught. And the emotional appeal messages which convey a group’s attitude towards behavior and informal social norms to consumers can be effective to reduce non-sustainable consumption behavior even when user behavior is difficult to monitor.

In addition, because there is no transfer of ownership in ABSs, consumer involvement becomes relatively weak, so they are reluctant to invest too much cognitive effort in the behavioral decision process (Zhang et al., 2020). Compared with emotional appeal messages, rational appeal messages contain more calculated content and requires consumers to invest more cognitive resources to understand them (Wu and Wang, 2011). Therefore, emotional appeal message is more in line with the cognition state of consumers in ABSs, and is more easily accepted by consumers.

Thus, we hypothesize:

*H1*: In ABSs, emotional appeal messages have a stronger persuasive effect on reducing non-sustainable consumption behavior than rational appeal messages.

### Effect of message framing on reducing non-sustainable consumption behavior

ABS firms can initiate pre-education or pre-cautionary vigilance by highlighting losses caused by non-sustainable consumption behavior or emphasizing the benefits of curtailing non-sustainable consumption behaviors.

On the one hand, due to the lack of actual ownership of products, consumers tend to pay more attention to their own interests than ABS firms in the process of using products. In terms of gains and losses, prospect theory focuses on the instinct of loss aversion (Kahneman and Tversky, 1979) and points out that consumers are generally more sensitive to loss-framed messages than gain-framed messages. Given an equal opportunity to attain gains or avoid losses, individuals are more inclined to avoid losses. The theory of negativity bias also postulates that, in the face of gains and losses of the same value, individuals have stronger perceptions of loss-framed messages and are more inclined to respond to negative messages (Baumeister et al., 2001; Seo and Park, 2019).

On the other hand, Lieberman et al. (2019) point out that punishments (losses) project stronger social norms and serve as stronger drivers of behavior than rewards (gains). In ABSs, consumers’ shared good usage behaviors are also influenced by perceived social norms (Schaefers et al., 2016a; Srivastava et al., 2021). Theoretically, loss-framed messaging is more indicative of the social norms of “not engaging in non-sustainable consumption behavior” than gain-framed messaging, thus making it easier to persuade consumers to reduce non-sustainable behavior.

Thus. we present the following hypothesis:

*H2*: In ABSs, loss-framed messages have a stronger persuasive effect on reducing non-sustainable consumption behavior than gain-framed messages.

### The interactive effect of message appeal and message framing

In the face of rational appeal messages, consumers are prone to initiate cognitive message processing and focus on the calculation of actual benefits (Wu and Wang, 2011). Based on loss aversion (Kahneman and Tversky, 1979) and the theory of negativity bias (Seo and Park, 2019), consumers are more sensitive to loss of benefits and are therefore more inclined to accept loss-framed rational appeal messages than gain-framed rational appeal messages.

When faced with emotional appeal messages, consumers differ in their cognitive response to positive and negative emotional information. Negative emotional information, which generally conflicts with consumers’ existing emotions, often evokes complex and comprehensive considerations involving individual cognitive resources combined with other relevant information (Manrai et al., 1997). This complicated consideration process leads to results in an unstable persuasive effect and may even create an opposite persuasive effect (Bessarabova et al., 2015). However, when given positive emotional information that is consistent with the individual’s current emotional state, the consumer usually does not use cognitive resources (Manrai et al., 1997). Furthermore, positive emotional appeal messages can trigger emotional and sensory pleasure (Fredrickson, 2001), which in turn allow the consumer to embrace the message. Meneses’s (2010) research also found that positive emotional information has more influence on consumers’ purchasing behavior than negative emotional information. Therefore, we posit that positive gain-framed emotional appeal messaging has a greater persuasive effect on reducing non-sustainable consumption behavior than negative loss-framed emotional appeal messaging. Thus, we formulate the following hypothesis:

*H3*: In ABSs, message appeal and message framing have interactive persuasive effects on reducing non-sustainable consumption behavior:

(a) when users receive rational appeal messages, loss-framed rational appeal messaging has a stronger persuasive effect on non-sustainable consumption behavior than gain-framed rational appeal messaging;
(b) when users receive emotional appeal messages, gain-framed emotional appeal messages have a stronger persuasive effect than loss-framed messages.

### Moderating effect of psychological ownership

ABS consumers do not have legal ownership of the shared goods they utilize, but only shared access to those goods (Lovelock and Gummesson, 2004; Bardhi and Eckhardt, 2012). However, long-term use of an item can engender a feeling of psychological ownership of the item (Strahilevitz and Loewenstein, 1998). Scholars have found that when individuals have a high level of psychological ownership of objects, certain citizenship behaviors are activated (Jussila et al., 2015; Lee et al., 2019). However, other scholars have found that when individuals have a high degree of psychological ownership of an item, they may develop a sense of territorialism (Brown et al., 2005) and react negatively to others who display ownership signals to the item (Kirk et al., 2018).

Compared with the emotional appeal messaging based on values recognition and emotional resonance, rational appeal messaging based on rules and policies establishes normative requirements for consumer behavior. In other words, users are required to use the goods provided by the firm in a specified manner. This kind of specification implies firm ownership of the goods. Furthermore, as control mechanisms, rules and policies are often viewed as restrictions on autonomy and free choice (Falk and Kosfeld, 2006), which may produce a negative response or even resistance from message recipients (Kirchler, 2007; Joffily et al., 2014). In addition, Shen and Dillard (2007) point out that loss-framed messages may be perceived as threatening and lead to defensive responses. Thus, individuals may reject loss-framed messages that they consider threatening (Van’t Riet and Ruiter, 2013).

When consumers have a high degree of psychological ownership of goods, they have a strong sense of control over the goods (Kirk et al., 2018). On the one hand, a loss-framed rational appeal message transmitting ownership signals may conflict with the sense of territorialism of consumers with high psychological ownership and lead to negative responses (Kirk et al., 2018). On the other hand, consumers may perceive that their agency is being restricted by the ABS firm in light of the requirements of the loss -framed rational appeal messaging. Reactance theory (Brehm, 1966) postulates that consumers employ resistance to retain their freedom of choice. In contrast, gain-framed emotional appeal messaging emphasizes incentives related to the benefits of behavioral change (Buyucek et al., 2019) rather than behavioral restrictions and does not signal the firm’s control over the shared goods. Seo and Park (2019) found that consumers were more inclined to accept a gain-framed message when their psychological ownership was high. Therefore, when psychological ownership is high, gain-framed emotional appeal messaging is more easily accepted by consumers.

When psychological ownership of shared goods is low, consumers do not have a sense of connection and control over the goods that leads to excessive interpretation or identification of ownership signaling in messaging (Kirk et al., 2018). Rather, they focus more on message content. Some scholars have suggested that when psychological ownership is low, individuals are more concerned about the loss of benefits (Seo and Park, 2019). Given that consumers associate loss-framed rational appeal messages with actual loss more than gain-framed emotional appeal messages, the persuasive effect of loss-framed rational appeal messaging is more effective. Thus we formulate the following hypothesis:

*H4*: In ABSs, the level of psychological ownership has a moderating effect on the persuasive effect of messages on behavior:

(a) When the consumers’ psychological ownership is high, the persuasive effect of gain-framed emotional appeal messaging on reducing non-sustainable consumption behavior is greater than loss-framed rational appeal messaging;
(b) When the consumers’ psychological ownership is low, the persuasive effect of loss-framed rational appeal messaging is greater than gain-framed emotional appeal messaging.

## Research methods

### Study overview

We tested our proposed conceptual framework (Supplementary Figure S1) and hypotheses (H1–H4) in the context of a car-sharing service, as this is one of the best known ABS services (Botsman and Rogers, 2010). A scenario-based design can increase realism by giving respondents a specific situation of non-sustainable consumption behavior. Using a scenario-based online experiment, we investigated the persuasive effect of messages on reducing non-sustainable consumption behavior in Study 1 and then tested the moderation effects of psychological ownership in Study 2.

### Study 1

#### Study design and respondents

A 2 × 2 two-factor completely random scenario-based online experiment was used to investigate the impact of message appeal (rational *vs*. emotional) and message framing (gain *vs*. loss) on reducing non-sustainable consumption behavior and the interactive effect of the two on reducing non-sustainable consumption behavior. Simultaneously, a control group that received no message was set up. There were a total of five randomized groups in this study.

We recruited 539 respondents from the customer pool of a popular MTurk-like website of China,^1^ which has over 2.6 million internet users from all over the country and was suitable for data collection (Jin et al., 2020). Due to the rapid development of the sharing economy in China, participants have knowledge about car-sharing. Among respondents, 220 were males and 319 were females. In terms of age distribution, 65 were 18–22 years old; 286 were 23–30 years old; 160 were 31–40 years old; and 28 were over 40. In terms of occupation distribution, 69 were students, 467 were in social professions, and 3 were unemployed.

#### Procedure

The experience of customer “A” when using a shared car was described to the respondents. Four different sets of experimental materials were provided. Each had two aspects of content manipulation. The first part was manipulation of the message appeal, and the second part was manipulation of the message framing.

First, we provided all respondents with basic information about car-sharing through text and picture information of the car (see Supplementary Figure S4). Second, after reading the basic information about car-sharing, pictures of random reminders were provided to the respondents, and they were told that “A” would see the same message on his mobile app page (see Supplementary Figure S5). The message materials are shown in Supplementary Table S1. In order to control influence factors, the vehicle type, picture location, plot of the story and reading time limit were exactly the same throughout the experiment. Respondents filled in the measurement questions after reading the materials.

In addition, Hawthorne effect has pointed out that people will deliberately change some behaviors when they realize that they are being watched or observed (McCambridge et al., 2014). Theoretically, we inferred that when consumers realized that their consumption behaviors were being watched by others, they may changed their intention of non-sustainable consumption behaviors. Therefore, we asked the respondent whether they thought that A’s behaviors would be observed by others, that is, “During the whole process of using the shared car, do you think anyone will observe the behaviors of ‘A’?” (1 = yes, 0 = no). And in this study, we named it as “perception of being observed” and used it as a control variable.

#### Manipulation check

Message content: A total of 98 respondents (64 females and 34 males) were recruited from a popular MTurk-like online research platform in China (see Footnote 1) and randomly assigned to one of four groups to evaluate different types of message content.

For message appeal, questions included: (1) I think the message is prompting me with emotional content; (2) I think the message is prompting me with rational content (7-point Likert scale, from 1 = strongly disagree to 7 = strongly agree). For message framing, questions included: (1) I think the message highlights the gain of the behavior; (2) I think the message highlights the loss of the behavior (7-point Likert scale, from 1 = strongly disagree to 7 = strongly agree). Because the survey items related to emotional messages and rational messages yielded opposite outcomes, we performed a reverse coding to calculate the mean, inverting the scales of the emotional messages items. We used a similar data analysis process for evaluation of the message framing.

Participants presented with rational messages exhibited a higher mean than those who read emotional messages (*M* rational = 4.554, SD = 1.473, *n* = 46 *vs*. *M* emotional = 3.212, SD = 1.230, *n* = 52, *t* = 4.917, *p* = 0.001). Respondents presented with gain-framed messages exhibited a lower mean than those who read loss-framed messages (*M* gain-framed = 3.064, SD = 1.159, *n* = 47 *vs*. *M* loss-framed = 4.853, SD = 1.474, *n* = 51, *t* = −6.640, *p* = 0.001). In conclusion, this study successfully manipulated the experimental material.

#### Variable measurement

Consumer misbehavior is the manifestation of non-sustainable consumption behavior in ABSs. The measurement of “non-sustainable consumption behavior” in this study refers to the measurement of “misbehavior intention” in the study of Schaefers et al. (2016a).

Because respondents are seldom willing to admit their own misbehavior (Fisher, 1993; Moody et al., 2018), this study infers respondents’ misbehavior intention by asking them to infer the behavior intention of “A” in the scenario. In the study of Schaefers et al. (2016a), questions include: After “A” picks up the shared car, do you think that: “‘A’ would not notify the car sharing company about a scratch he made in the car?”; “‘A’ would not notify the car sharing company if he slightly damaged the side mirror?,” “‘A’ would treat the car in a way that others may find unacceptable?” (Cronbach’s alpha = 0.85; Reliability = 77.66%). A 7-point Likert scale was used, ranging from 1 = very unlikely to 7 = very likely, to rate all items.

#### Data analysis results

In this study, SPSS24.0 was used to analyze variances in the collected data. Supplementary Table S2 shows the descriptive statistics of respondents’ non-sustainable consumption behavior intention.

First, results of one-way ANOVA on reducing non-sustainable consumption behavior intention show that the main effect of message appeal was significant, *F*(2, 536) = 3.606, *p* = 0.028.

Results of *post hoc* multiple comparisons show that the non-sustainable consumption behavior intention of respondents who received emotional appeal messages was lower than the control group that received no message (Diff = 0.401, *p* = 009), as shown in Supplementary Table S3. There was no difference in non-sustainable consumption behavior intention between respondents who received rational appeal messages and the control group (Diff = 0.115, *p* = 0.456). These results support H1.

Second, results of one-way ANOVA on reducing non-sustainable consumption behavior intention show that the main effect of message framing was significant, *F*(2, 536) = 3.634, *p* = 0.027. Results of *post hoc* multiple comparisons, as shown in Supplementary Table S3, indicate that the non-sustainable consumption behavior intention of respondents who received a loss-framed message was significantly lower than that of the control group that received no message (Diff = 0.402, *p* = 0.009), and the non-sustainable consumption behavior intention of respondents who received a gain-framed message was no different from that of the control group that received no message (Diff = 0.113, *p* = 0.464; see Supplementary Table S3). The results support H2.

To verify H3, we performed a two-way ANOVA, and the results indicate that the two-way interaction between message appeal and message framing are statistically significant, as shown in Supplementary Table S4, *F*(1, 534) = 15.208, *p* = 0.001. And according to the standard proposed by Cohen (1988), this interaction effect size is small (see Supplementary Table S4). Furthermore, as shown in Supplementary Table S5, the interaction effect was still significant even when we included “perception of being observed” as a covariate.

As Supplementary Figure S2 shows, when receiving rational appeal messaging (*M* loss-framed rational = 3.907, *M* gain-framed rational = 4.785, *M* no message = 4.461, *F*(2, 356) = 8.980, *p* = 0.001), the intention of the control group which received no message to engage in non-sustainable consumption behavior was significantly higher than the group that received loss-framed rational appeal messaging (Diff = 0.554, *p* = 0.003), and the control group’s intention to engage in non-sustainable consumption behavior was marginally lower than the group that received gain-framed rational appeal messaging (Diff = −0.324, *p* = 0.081). The results support H3a.

When receiving emotional appeal messaging (*M* loss-framed emotional = 4.211, *M* gain-framed emotional = 3.906, *M* no message = 4.461, *F*(2, 356) = 4.709, *p* = 0.01), respondents’ non-sustainable consumption behavior intention in the control group was significantly higher than in the group that received gain-framed emotional appeal messaging (Diff = 0.555, *p* = 0.003), but there was no difference between the control group and the group that received loss-framed emotional appeal messaging (Diff = 0.250, *p* = 0.169). The results support H3b.

### Study 2

#### Study design and respondents

A 2 × 2 × 2 three factors completely random experiment was conducted to examine the moderating effect of psychological ownership (high *vs*. low) on the effect of message appeal (rational *vs*. emotional) on reducing non-sustainable consumption behavior and the moderating effect of psychological ownership on the interaction of message appeal and message framing (loss *vs*. gain) on reducing non-sustainable consumption behavior. At the same time, in the case of respondents’ different levels of psychological ownership (high *vs*. low), control groups that received no message were set up. There are a total of 10 randomized experimental groups in this study.

This study was conducted with respondents recruited in Study 1, and they have knowledge about car-sharing. And the respondents in Study 1 were asked to judge the length of time that “A” used the shared car this time.

#### Procedure

First, the content of message appeal and message framing was the same as in Study 1.

Second, referring to the research of Strahilevitz and Loewenstein (1998), we distinguished respondents with high and low psychological ownership. Strahilevitz and Loewenstein (1998) note in their research that consumers develop psychological ownership after long-term use of a good. Therefore, in this experiment, we first asked respondents to guess how long “A” would use the shared car. Then respondents were asked to talk about their perception of A’s duration of access. Questions to the respondents included: “How long do you think A will use the car this time? Do you think the time you mentioned is relatively long or relatively short?” If the participant indicates that their perception of A’s access duration is long, it suggests that the participant’s psychological ownership is relatively high, whereas if the participant indicates that their perception of A’s access duration is short, it suggests that the participant’s psychological ownership is relatively low.

In addition, we asked respondents whether they thought that A’s behaviors would be observed by others.

#### Manipulation check

In order to verify the direct relationship between perceived use time and psychological ownership, the measurement of psychological ownership by Fuchs et al. (2010) was used to ask respondents whether they agree with the following questions: “Although ‘A’ does not legally own the car yet, he has the feeling that the car is him”; “‘A’ may feel that this car belongs to him”; “‘A’ may feel that this car is part of him”; and so on (Cronbach’s alpha = 0.84; Reliability = 75.94%).

The results show that when the perception of access duration is short, the level of psychological ownership of the shared car is significantly lower than the psychological ownership of respondents whose perception of access duration is long (*M* short = 3.418, SD = 1.152, *n* = 47; *M* long = 4.135, SD = 1.376, *n* = 47; *p* = 0.02).

#### Variable measurement

The measurement of “non-sustainable consumption behavior” is consistent with Study 1. This study measured respondents’ “misbehavior intention” (Cronbach’s alpha = 0.85; Reliability = 77.66%; Schaefers et al., 2016a).

#### Data analysis results

As shown in Supplementary Tables S6, S7, message appeal, message framing, and consumers’ psychological ownership have no interactive impact on non-sustainable consumption behavior. But interestingly, gain-framed emotional appeal messages and loss-framed rational appeal messages have a significant interaction effect with psychological ownership (see Supplementary Table S8). And according to Cohen (1988) standard, this interaction effect size is small. Meanwhile, as shown in Supplementary Table S9, the interaction effect was still significant even when the “perception of being observed” was included as a covariate.

Descriptive statistics regarding consumers’ non-sustainable consumption behavior intention are provided in Supplementary Table S10. Results of *post hoc* multiple comparisons, as shown in Supplementary Figure S3, when consumers’ psychological ownership of the goods is high (*M* loss-emotional = 3.967, *M* gain-emotional = 3.752, *M* loss-rational = 4.272, *M* gain-rational = 4.899, *M* no message = 4.326, *F*(4, 254) = 3.788, *p* = 0.005). As shown in Supplementary Figure S2, the non-sustainable consumption behavior intention of respondents who received a gain-framed emotional appeal message was significantly lower than those who received no message (*M* no message = 4.326, *M* gain-emotional = 3.752, Diff = 0.574, *p* = 0.048). However, there was no significant difference between those who received loss-framed emotional appeal messaging and the control group (*M* no message = 4.326, *M* loss-emotional = 3.967, Diff = 0.359, *p* = 0.190). There was no significant difference between the loss-framed rational appeal message group and the control group (*M* no message = 4.326, *M* loss-rational = 4.272, Diff = 0.054, *p* = 0.835). And the control group was significantly lower than the group receiving gain-framed rational appeal messaging (*M* no message = 4.326, *M* gain-rational = 4.899, Diff = −0.571, *p* = 0.030). In summary, the results support H4a. That is, when the consumers’ psychological ownership is high, only gain-framed emotional appeal messaging can reduce their non-sustainable consumption behavior intention.

Furthermore, when consumers’ psychological ownership of goods was low (*M* loss-emotional = 4.415, *M* gain-emotional = 4.006, *M* loss-rational = 3.472, *M* gain-rational = 3.667, *M* no message = 4.591, *F*(4, 275) = 5.907, *p* = 0.001), as shown in Supplementary Figure S2, the non-sustainable consumption behavior intention of respondents who received gain-framed emotional appeal messaging was significantly less than those who did not receive a message (*M* no message = 4.591, *M* gain-emotional = 4.006, Diff = 0.585, *p* = 0.016). However, there was no significant difference between those who received loss-framed emotional appeal messaging and the control group (*M* no message = 4.591, *M* loss-emotional = 4.415, Diff = 0.176, *p* = 0.483). Meanwhile, the loss-framed rational group was lower than the control group (*M* no message = 4.591, *M* loss-rational = 3.472, Diff = 1.119, *p* = 0.001). And there was no difference between respondents who received the gain-framed rational appeal message and the respondents who did not receive a message (*M* no message = 4.591, *M* gain-rational = 4.667, Diff = −0.076, *p* = 0.769). The results indicate that both loss-framed rational appeal messaging and gain-framed emotional appeal messaging can persuade consumers to reduce non-sustainable consumption behavior when psychological ownership is low. Moreover, it is worth noting that the persuasive effect of loss-framed rational appeal messaging is marginally stronger than that of gain-framed emotional appeal messaging at this time (*M* loss-rational = 3.472, *M* gain-emotional = 4.006; *p* = 0.069). This result supports H4b. That is, when psychological ownership is low, loss-framed rational appeal messaging is better than gain-framed emotional appeal messaging at persuading consumers to reduce non-sustainable consumption behavior.

## General discussion

### Findings

In view of the prevalence non-sustainable consumption behaviors in ABSs, platform managers often try to manage users in advance through message strategies. This study investigates the persuasive effects of different messaging strategies on curtailing users’ non-sustainable consumption behaviors and examines the moderating effect of psychological ownership.

These findings demonstrate that messaging strategy can serve as an effective management approach to reducing non-sustainable consumption behavior. Both the message appeal strategy (rational *vs*. emotional) and the message framing strategy (loss *vs*. gain) have persuasive effects on reducing non-sustainable consumption behavior, and there is an interactive effect between the two. In ABSs, due to the relatively weak role of process governance and post-event feedback, the deterrence effect of rules is weak, and thus the persuasive effect of rational appeal messaging on reducing non-sustainable consumption behavior is lower than emotional appeal messaging. This result is consistent with some scholars’ conclusions that emotional appeal messaging is more effective (Lwin et al., 2014; De Veirman et al., 2015; Zhang et al., 2020). At the same time, similar to research in traditional consumption scenarios, such as vaccinations and healthy diet (Wirtz and Kulpavaropas, 2014; Liu et al., 2019), loss-framed messages can be more persuasive to reduce non-sustainable consumption behavior than gain-framed messages.

More interestingly, our research found that when ABS consumers received rational appeal messages, loss-framed rational appeal messaging had a stronger persuasive effect on unsustainable consumption behavior than gain-framed rational appeal messaging. The possible reason for this is that consumers are more inclined to accept loss-framed rational appeal messages than gain-framed rational appeal messages because of loss aversion (Kahneman and Tversky, 1979) and negativity bias (Seo and Park, 2019). However, when it came to emotional appeal messages, the result was the opposite. Gain-framed emotional appeal messaging had a stronger persuasive effect on reducing non-sustainable consumption behavior intention than the loss-framed messaging. Specifically, compared with loss-framed emotional appeal messaging, gain-framed emotional appeal messaging is usually consistent with an individual’s current emotional state, and does not cause consumers to use cognitive resources and make complex and comprehensive considerations about the message (Manrai et al., 1997). As a result, the persuasive effect of gain-framed emotional appeal messaging on consumers behavior is more stable (Bessarabova et al., 2015). And gain-framed emotional appeal messaging is also more likely to trigger emotional and sensory pleasure than loss-framed emotional appeal messaging (Fredrickson, 2001) and promotes consumers to accept the message.

ABSs users do not have legal ownership of goods (Fritze et al., 2020), but certain usage conditions can trigger various forms of psychological ownership with respect to the goods (Strahilevitz and Loewenstein, 1998), which may influence the messaging’s effect on non-sustainable consumption behavior. First, consumers’ psychological ownership of goods affects the persuasive effect of messages. This study found that when psychological ownership is high, only gain-framed emotional appeal messages could effectively reduce non-sustainable consumption behavior. One possible explanation is that high psychological ownership imparts a sense of control and territoriality over shared goods (Kirk et al., 2018). While users may resist the control of ABSs firms, when ownership signals displayed by rational appeal messaging trigger a territorial response, it reduces their acceptance of the rational appeal message. In contrast, gain-framed emotional appeal messages place more emphasis on incentives related to the benefits of behavioral change (Buyucek et al., 2019), so they are more likely to be accepted by consumers with high psychological ownership. When psychological ownership is low, without the territorial response of high psychological ownership, both loss-framed rational appeal and gain-framed emotional appeal messages can effectively persuade users to reduce non-sustainable consumption behavior. In addition, our results indicate that the persuasive effect of loss-framed rational appeal messaging on reducing non-sustainable consumption behavior is greater than that of gain-framed emotional appeal messaging, which indicates that in the context of low psychological ownership, consumers still pay more attention to their actual losses (Seo and Park, 2019). In ABSs, given that access to and ownership of shared goods are separate and there are differences in usage time, platform users inevitably have different levels of psychological ownership. Therefore, it is necessary to conduct further research on consumers’ psychological ownership in the future.

### Theoretical implications

Previous research on green marketing has primarily focused on promoting sustainable consumption behaviors; however, this study focuses on how to inhibit non-sustainable consumption behaviors.

First, this study advances research on the motivation for non-sustainable consumption behaviors in ABSs into governance research, providing a useful tool for scholars to further study this field. Given the need for non-sustainable consumption behavior management in ABSs (Srivastava et al., 2021), this study explores the persuasive effects of different messaging strategies on reducing consumers’ non-sustainable consumption behaviors from the perspective of pre-behavior governance. Based on various message appeals and message framings, our study categorizes messaging strategies as message appeal strategies and message framing strategies. Based on this, we analyze the different effects of the two messaging strategies on the non-sustainable consumption behavior of platform users. This is different from extant literature that focuses more on analysis of the causes of unsustainable behavior (Schaefers et al., 2016a; Jia et al., 2018; Srivastava et al., 2021). We are more concerned with strategies to manage non-sustainable consumption behavior and the impact of consumers’ psychological ownership on strategy effectiveness. We believe that this provides a good basis for scholars to further develop consumer behavior research in this domain.

Second, this study expands research on the impact of messaging strategies on individual behavior. On the one hand, our study introduces a new behavioral scope for message persuasion effects. That is, it explores the persuasive effect of messaging strategies based on different message appeals (emotional *vs*. rational) and message framings (loss *vs*. gain) on constraining non-sustainable consumption behavior in ABSs, and further explores the interactive persuasive effect of the two messaging strategies. On the other hand, this study enhances research on the relationship between message framing and psychological ownership and supplements the conclusions of Seo and Park (2019). In ABSs, the separation between ownership of goods and access is obvious, which makes clear the disconnect between rational appeal messaging that uses ownership signals and the high psychological ownership of consumers and further invalidates the persuasive effect of rational appeal messaging. Our study results show that when consumers have high psychological ownership, only gain-framed emotional appeal messages can persuade them to reduce non-sustainable consumption behaviors in ABSs. When consumers have low psychological ownership, both loss-framed rational appeal and gain-framed emotional appeal messages can effectively persuade them to eschew non-sustainable consumption behaviors. Furthermore, the persuasive effect of loss-framed rational appeals on reducing non-sustainable consumption behavior is greater than that of gain-framed emotional appeal messages. This finding shows that psychological ownership has a significant moderating effect on ex-ante governance messaging strategies with regard to behavior in ABSs scenarios. Therefore, the role of psychological ownership in ABSs needs further research and development.

Third, this study enriches research on message “nudge” in behavioral economics, in which scholars propose that messaging can serve as a “nudge” to induce consumers to engage in certain behaviors without prohibiting any choices or significantly altering their economic incentives (Thaler and Sunstein, 1999). There has been research and practice management demonstrating the behavioral efficacy of message nudges, such as the use of messages to promote desirable health behaviors (Bavel et al., 2020; United Nations Development Program, 2020), government anti-smoking education campaigns (Sunstein, 2016), and others. This study refers to these viewpoints and applies them to ABS behavioral research. Our research found that the targeted use of some information cues can indeed have the expected “nudge” effect on individual use behavior in ABSs, such that gain-framed emotional appeal messages can persuade consumers to reduce non-sustainable consumption behavior without directly altering individual economic motivations. Similar to the perspective of previous studies focusing on how psychological factors affect individuals’ support for “nudges” (Jung and Mellers, 2016), this study explores the impact of users’ psychological ownership on the persuasive effect of messages. The results show that gain-framed emotional appeal messaging is more effective at persuading individuals with high psychological ownership.

### Practice implications

ABS consumers are prone to non-sustainable consumption behaviors because of the ability to conceal and the externality of such behaviors. Sustainable development of this industry depends on reducing such behaviors.

The findings of this study point to an easy and cost-effective management method that employs messaging strategies to persuade consumers to constrain non-sustainable consumption behaviors. Specifically, firms can use platforms’ mobile apps or new media information channels to push gain-framed emotional appeal or loss-framed rational appeal messages to consumers to inhibit non-sustainable consumption behaviors.

Second, the use of gain-framed emotional appeal or loss-framed rational appeal messages also needs to take into account the consumers ${}^{’}\;$ level of psychological ownership. This study demonstrates that the level of psychological ownership of shared goods affects platform consumers’ message acceptance. When psychological ownership is high, only an gain-framed emotional appeal has a persuasive effect on non-sustainable consumption behaviors. Additionally, measuring consumers’ openness to psychological ownership using initial questions in the mobile app may enable firms to identify appropriate persuasive messaging strategies.

Third, the results of this study have significance for public administrators. Shared goods absorb public resources. For example, shared bikes park on public land. Public communication with gain-framed emotional appeal messages can effectively reduce non-sustainable consumption behaviors with regard to shared goods. Curtailing non-sustainable consumption behaviors reduces waste of public resources and lowers management costs for public management departments, thereby abetting the sustainable development of society.

### Limitations and future research

This research still has some deficiencies in the research process and and there are more researches worth exploring in the future.

First, research sample is still limited. There are many industries involved in the ABSs. This research is only based on a car-sharing scenario, which is one of the most common shared services in the sharing economy (Botsman and Rogers, 2010). Future research may explore the impact of message appeal and message framing on non-sustainable consumption behaviors in different contexts, such an P2P accommodation, etc.

Second, the research method of this study limits the reflection of the actual behavioral intentions of the experimental participants, and the actual behavioral differences may be even greater. Although in the experiment of this research, the possibility of non-sustainable consumption behavior of the participants was reflected by asking the participants about their guesses about the possibility of non-sustainable consumption behavior of the protagonist of the experiment material. However, considering that individuals will actively maintain their self-image, the intention of the respondents’ non-sustainable consumption behavior may be lower than actual behavior result. In order to further verify the results of this study, effective field study can be designed for verification in the future.

Third, this research does not further examine the mediating mechanisms by which messaging affects non-sustainable consumption behaviors. Future research can explore the psychological mechanism of which messaging persuades consumers to reduce non-sustainable consumption behaviors.

Fourth, considering multiple consumers successively gain temporal access to a good in ABSs (Schaefers et al., 2016a), current non-sustainable consumption behaviors will impair the normal use of subsequent consumers (Belk, 2010). Future research may explore other message appeals, such as self-interest appeals and altruism appeals (Green and Peloza, 2014), with a view to providing additional context for messaging strategies.

## Data availability statement

The original contributions presented in the study are included in the article/Supplementary material, further inquiries can be directed to the corresponding author.

## Ethics statement

Ethical review and approval was not required for the study on human participants in accordance with the local legislation and institutional requirements. Written informed consent for participation was not required for this study in accordance with the national legislation and the institutional requirements.

## Author contributions

YX wrote and revised the manuscript under the guidance of the supervisor XF. All authors contributed to the article and approved the submitted version.

## Funding

This work was financially supported by the National Natural Science Foundation of China (71672150).

## Conflict of interest

The authors declare that the research was conducted in the absence of any commercial or financial relationships that could be construed as a potential conflict of interest.

## Publisher’s note

All claims expressed in this article are solely those of the authors and do not necessarily represent those of their affiliated organizations, or those of the publisher, the editors and the reviewers. Any product that may be evaluated in this article, or claim that may be made by its manufacturer, is not guaranteed or endorsed by the publisher.

## Supplementary material

The Supplementary material for this article can be found online at: https://www.frontiersin.org/articles/10.3389/fpsyg.2022.984222/full#supplementary-material

## Footnotes

^1^ www.wjx.com
