# Supplementary material for: Ex-ante reminders: The effect of messaging strategies on reducing non-sustainable consumption behaviors in access-based services
Source: Front Psychol. 2022 Sep 6;13:984222. doi: 10.3389/fpsyg.2022.984222 (PMC9486457; doi:10.3389/fpsyg.2022.984222)
Supplement: Supplementary file 1 [file Image_1.pdf]

Appendix A

As shown in Figure 1, participants can read a basic information about car-sharing.

Car-sharing refers to a model of car rental where people rent cars for short periods of time, which means that they can get access to a car without actually owning it. The procedure for picking up a car is quite simple. Scan the QR code on the car through your mobile app, then you can unlock the shared car.

“A” is a registered member of a car sharing company. Now “A” is going to use the shared car through the company’s mobile app.

The condition of the car is shown in the picture:

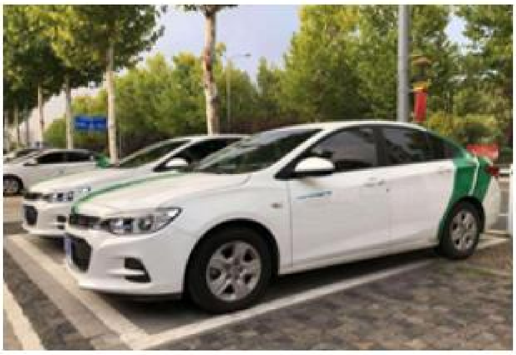

Figure 1 Car-sharing

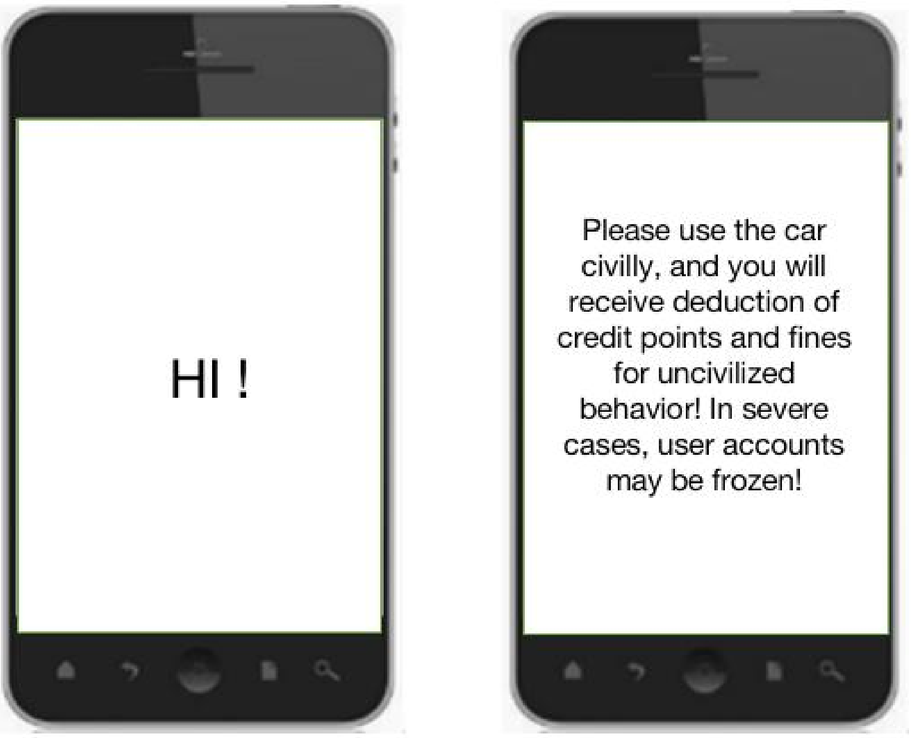

No Messaging | Messaging

Figure 2 Messaging strategies (example)
